# Delineating the trajectories and normative values of multi-systemic aging metrics in the UK, the US, and China

**DOI:** 10.1093/geroni/igaf122.2667

**Published:** 2025-12-31

**Authors:** Liming Zhang, Jiening Yu, Xueqing Jia, Jingyun Zhang, Wei Yang, Xi Chen, Emiel O Hoogendijk, Zuyun Liu

**Affiliations:** Zhejiang University, Hangzhou, Zhejiang, China (People’s Republic); Zhejiang University, Hangzhou, Zhejiang, China (People’s Republic); Zhejiang university, Hangzhou, Zhejiang, China (People’s Republic); Zhejiang University, Hangzhou, Zhejiang, China (People’s Republic); King’s College London, London, England, United Kingdom; Yale University, New Haven, Connecticut, United States; VU University Medical Center, Amsterdam, Noord-Holland, Netherlands; Zhejiang University, Hangzhou, Zhejiang, China (People’s Republic)

## Abstract

Aging is a multi-dimensional process and manifests heterogeneities across different organ systems, individuals, and countries. Here we used three large national datasets (UKB, NHANES, and CHARLS) to delineate the trajectories of 14 organ/system-representative aging metrics for persons under different sociodemographic contexts in the UK, the US, and China. The functions of all aging metrics, except for cognitive function, manifested a progressive deterioration or maintained stability after adulthood, especially after middle age. The deterioration of cognitive function was more pronounced after the age of 70 in Chinese and British populations, while not in the American populations. In the stratified analyses, males and females manifested disparities in aging trajectories involving the cardiovascular and muscle-skeletal systems. Notably, we observed substantial disparities in the aging trajectories by economic and educational levels within and between the three countries: the low-income and low-education subgroups in China manifested a more pronounced deterioration in the functioning of multiple systems (i.e., brain, muscle-skeletal, physical function, and pulmonary). However, these income or educational disparities were nonexistent in the UK and the US participants, implying health inequalities caused by regional socioeconomic disparities. This work may serve as a proof-of-concept to comprehend the multi-dimensional signature of systemic aging and call for policies to promote health equity across nations when facing dramatic global aging.

